# MASTL: A novel therapeutic target for Cancer Malignancy

**DOI:** 10.1002/cam4.3141

**Published:** 2020-07-21

**Authors:** Iram Fatima, Amar B. Singh, Punita Dhawan

**Affiliations:** ^1^ VA Nebraska‐Western Iowa Health Care System Omaha NE USA; ^2^ Department of Biochemistry and Molecular Biology University of Nebraska Medical Center Omaha NE USA; ^3^ Buffet Cancer Center University of Nebraska Medical Center Omaha NE USA

**Keywords:** CANCER, CELL cycle, chemoresistance, MASTL

## Abstract

Targeting mitotic kinases is an emerging anticancer approach with promising preclinical outcomes. Microtubule‐associated serine/threonine kinase like (MASTL), also known as Greatwall (Gwl), is an important mitotic kinase that regulates mitotic progression of normal or transformed cells by blocking the activity of tumor suppressor protein phosphatase 2A (PP2A). MASTL upregulation has now been detected in multiple cancer types and associated with aggressive clinicopathological features. Apart, an aberrant MASTL activity has been implicated in oncogenic transformation through the development of chromosomal instability and alteration of key oncogenic signaling pathways. In this regard, recent publications have revealed potential role of MASTL in the regulation of AKT/mTOR and Wnt/β‐catenin signaling pathways, which may be independent of its regulation of PP2A‐B55 (PP2A holoenzyme containing a B55‐family regulatory subunit). Taken together, MASTL kinase has emerged as a novel target for cancer therapeutics, and hence development of small molecule inhibitors of MASTL may significantly improve the clinical outcomes of cancer patients. In this article, we review the role of MASTL in cancer progression and the current gaps in this knowledge. We also discuss potential efficacy of MASTL expression for cancer diagnosis and therapy.

## Graphical abstract

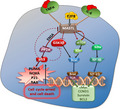

## 1 INTRODUCTION

Cancer is a leading cause of morbidity and mortality throughout the world, accounting for an estimated 9.6 million deaths in 2018.^1^ Cancer cells have the ability to develop resistance to traditional therapies and there is an increasing prevalence of resistant cancers therefore, further research to develop new treatments for cancer is necessary. Furthermore, improved understanding of the molecular mechanism of carcinogenesis is important for the cancer prevention, its early diagnosis and improved prognosis. Elucidation of the relevant cellular pathways that render cancer cells to become therapeutically resistant will expedite the development of cancer specific therapeutics.

Importantly, a distinctive feature of malignant cancer comprises abnormal proliferation of cancer cells, which interferes with the normal function of surrounding or distant tissues (in case of metastasis); a leading cause of cancer‐related deaths.^2^ Cell division comprises a series of well‐coordinated events and includes the equal distribution of replicated DNA and cellular components into two daughter cells.^3^ Cell cycle checkpoints are surveillance mechanism/s that function to monitor and maintain the proper execution of cell cycle processes.^4^, ^5^ Chromosomal instability (CIN) or genetic instability increases genomic mutation rate and is associated with oncogenic transformation. This is acquired predominantly through the abrogation of cell cycle checkpoints.^6^, ^7^ Accordingly, cell cycle regulators such as cyclin‐dependent kinases (CDKs), Polo‐like kinase 1 (PLK1), and Aurora kinases have emerged as important mitotic regulators for the maintenance of chromosomal stability and cell cycle progression.^8^, ^9^, ^10^, ^11^, ^12^, ^13^ Moreover, these cell cycle regulatory kinases are overexpressed in many cancer types^9^, ^14^ and multiple small molecule inhibitors targeting theses kinases are currently undergoing clinical evaluation for the treatment of cancer.^14^, ^15^, ^16^

In recent years, microtubule‐associated serine/threonine kinase like (MASTL) has gained attention in the regulation of cellular mitosis. Originally, MASTL or Greatwall (Gwl) was discovered in the Drosophila as an essential kinase required for the correct chromosome condensation and cell cycle progression through mitosis and meiosis.^17^, ^18^, ^19^, ^20^ The role of MASTL in regulating mitosis is now well‐defined.^20^, ^21^, ^22^ Also, a key role of MASTL in oncogenesis has recently been proposed in different cancer types,^23^, ^24^, ^25^, ^26^ however, details of the underlying mechanism/s and/or factors regulating MASTL expression/activity during cancer progression remain unclear and needs detailed molecular investigation. In the light of the critical role of MASTL in cancer progression and unclear knowledge of its cancer promoting role and regulation, we present this review article that summarizes the knowledge from the recent publications regarding the role of MASTL deregulation in cancer progression, mechanism/s by which MASTL promotes tumorigenesis and its efficacy as a novel anticancer therapeutic target.

## 2 THE ROLE OF MASTL IN MITOSIS

Although regulation of mitosis is complex, several studies have demonstrated that the activation of the cyclin B1‐Cdk1 complex triggers cell mitosis by promoting nuclear envelope breakdown, chromosome condensation, and spindle assembly.^27^, ^28^, ^29^, ^30^ At the G2 phase of the cell cycle, the inhibitory phosphorylation pathway is active and cyclin B1‐Cdk1 complex is kept in an inactive state by phosphorylation on Cdk1 at T14 and Y15 by Myt1 and Wee1 kinases, respectively.^31^, ^32^, ^33^ During G2/M transition phase, theses kinases become inactive, whereas cell division cycle 25 (Cdc25) becomes phosphorylated and active^34^ which leads to dephosphorylation of the inhibitory residue and promotes cyclin‐B–Cdk1 activation and mitotic entry.^33^, ^35^, ^36^ MASTL is an important kinase for the progression of mitosis and maintenance of mitotic state by inhibiting PP2A‐B55, a protein phosphatase that antagonizes the effects of cyclin B–Cdk1.^37^, ^38^, ^39^ MASTL acts as a regulator of mitotic progression through the phosphorylation of α‐endosulfine (ENSA) and/or cAMP‐regulated phosphoprotein 19 (ARPP19), which subsequently inhibits the activity of protein phosphatase 2A complex (PP2A‐B55).^21^, ^40^, ^41^, ^42^ Thus, inhibition of PP2A‐B55 is essential for the maintenance of cyclin B1‐Cdk1 activity during normal mitosis.^40^, ^43^, ^44^, ^45^, ^46^

Two independent studies identified two unique substrates of MASTL: the small and unstructured proteins ARPP19 and ENSA.^44^, ^45^ These two proteins ARPP19 and ENSA are highly homologous and their phosphorylation by MASTL at a serine residue (S62 and S67, respectively) promotes their binding to PP2A‐B55 holocomplex and inhibiting it, which results in mitotic entry.^44^, ^45^ Furthermore, a key study by Hached et al using conditional knockout mouse models, demonstrated that Arpp19 is essential for embryonic development and Arpp19 ablation results in dramatic mitotic defects due to the premature dephosphorylation of proteins involved in DNA condensation (Capd3), cytokinesis (PRC1), and nuclear pore reformation (NUPs and Lamins A/C).^47^ Interestingly, besides MASTL‐dependent phosphorylation, ARPP19, and ENSA are also phosphorylated by other kinases such as by PKA (S104 and S109) and by Cdk1 (T28).^44^ Phosphorylation of ENSA at the different sites had qualitatively and/or quantitatively different effects on PP2A–B55 inhibition suggesting ARPP19/ENSA functions as a “stepwise tuner” for PP2A–B55. When AARP19 is phosphorylated by PKA at S109, it restrains Cdk1 activation while when phosphorylated by Greatwall at S67, ARPP19 becomes an inducer of Cdk1 activation.^39^, ^44^

The inhibitory function of MASTL is required to prevent mitotic collapse, whereas inhibition of MASTL and reactivation of PP2A is required to trigger mitotic exit.^48^, ^49^ In this regard, knockdown of MASTL expression in HeLa cells promoted substantial mitotic defects, including chromosome misalignment, mis‐segregation, and severe cytokinesis defects.^21^, ^50^ Further investigation revealed improper dephosphorylation of the cyclin B1‐Cdk1 substrates since they were fully rescued by the chemical inhibition of the phosphatase PP2A.^21^, ^50^, ^51^ In addition, by promoting dephosphorylation of nuclear pore complex protein (NUP153), MASTL is essential for nuclear pore reformation and subsequent recruitment to chromatin.^42^ Overall, these data provide strong support for the role of MASTL in controlling PP2A‐B55 activity to regulate mitosis progression through the phosphorylation of several important substrates which are associated with anaphase entry, cytokinesis, and nuclear pore reformation.

## 3 THE ROLE OF MASTL IN CELLULAR TRANSFORMATION AND ONCOGENIC SIGNALING PATHWAYS

Aberrant activity of cell cycle kinases is frequently associated with cancer cells and therefore these regulatory kinases act as potential biomarkers of proliferation and attractive druggable target for future anticancer therapies. Several studies have reported that MASTL is highly expressed in a variety of human cancers. Furthermore, increased MASTL expression has been associated with poor outcomes in breast, oral, gastric, colon, and head and neck cancer, suggesting that MASTL plays a master role in carcinogenesis.^24^, ^25^, ^26^, ^49^, ^52^, ^53^, ^54^ Disrupting the MASTL‐ENSA‐PP2A‐B55 (MEP) axis results in multiple mitotic errors^21^, ^50^, ^55^, ^56^ which then drive CIN, a hallmark of cancer.^57^ Recent studies have also demonstrated that MASTL promotes oncogenesis and therapy resistance in cancer cells by enhancing oncogenic AKT kinase activity^54^ and Wnt signaling.^24^ Moreover, MASTL upregulation was associated with recurrence after initial treatment, thereby decreasing cancer patient survival in a number of cancer types.^24^, ^52^, ^53^, ^58^

Similar studies in breast cancer have demonstrated that MASTL expression correlates significantly with increased CIN, mitotic index, histological grade, poor overall survival and with a high risk of metastatic relapse in estrogen receptor (ER) positive patients.^25^, ^49^, ^52^, ^59^ Overexpression of wild‐type MASTL in immortalized human MCF10A breast epithelial cells was sufficient to increase the rate of chromosome bridges, micronuclei formation as well as to induce loss of contact inhibition,^25^, ^54^ whereas inhibition of MASTL selectively killed breast cancer cells by induction of mitotic catastrophe.^52^ Other than its effects on mitosis, MASTL promotes oncogenesis by activating AKT kinase activity via degradation of its phosphatase, PH (pleckstrin homology) domain Leucine‐rich repeat Protein Phosphatase (PHLPP),^54^ regulates normal DNA replication timing^60^ and recovery from the premitotic DNA damage checkpoint arrest.^61^ Overall, upregulation of MASTL expression induces partial epithelial to mesenchymal transition (EMT), abnormal proliferation growth, as well as disrupts the timing of mitotic exit, increased chromosome segregation defects and micronuclei formation.^25^, ^26^ In 42.9% of gastric cancer patients, MASTL was significantly associated with cancer metastasis, tumor relapse, and poor overall survival, suggesting the potential of MASTL expression as a valuable prognostic marker and a potential therapeutic target for patients with gastric cancer.^26^ Similarly, Cetti et al identified MASTL as an important target for thyroid tumor cells.^62^ In this study, MASTL was identified as the top gene among a list of genes implicated for their potential in inducing the growth of several thyroid tumorcell lines.^62^ Depletion of MASTL associated with mitotic catastrophe and increased levels of DNA damage and cell death, and thus enhanced the sensitivity to cisplatin treatment. Yet another study by Cao et al have shown a pivotal role of MASTL in the development of chronic hepatitis‐associated liver cancer.^63^ The upregulated expression of MASTL is associated with attenuated DNA damage signaling and apoptotic response^53^ In previous studies, it was demonstrated that depletion of MASTL from interphase *Xenopus* egg extracts resulted in elevated DNA damage signaling and impeded checkpoint recovery.^61^ In response to DNA damage, cells stimulate complex signaling cascades which includes execution of DNA repair, the activation of cell cycle checkpoints and initiation of apoptosis, and is therefore critically involved in cancer progression and therapy.^64^ Moreover, It has also been shown that MASTL expression promotes recovery from DNA damage and inhibiting MASTL has been demonstrated to be beneficial for DNA damage‐based therapies.^65^ However, MASTL also regulates cell cycle in normal cells and MASTL deficient mice die early in development.^22^ Therefore, to further define the role MASTL as a therapeutic, some of the conclusions still remain to be validated and future studies will address these issues.

**FIGURE 1:**
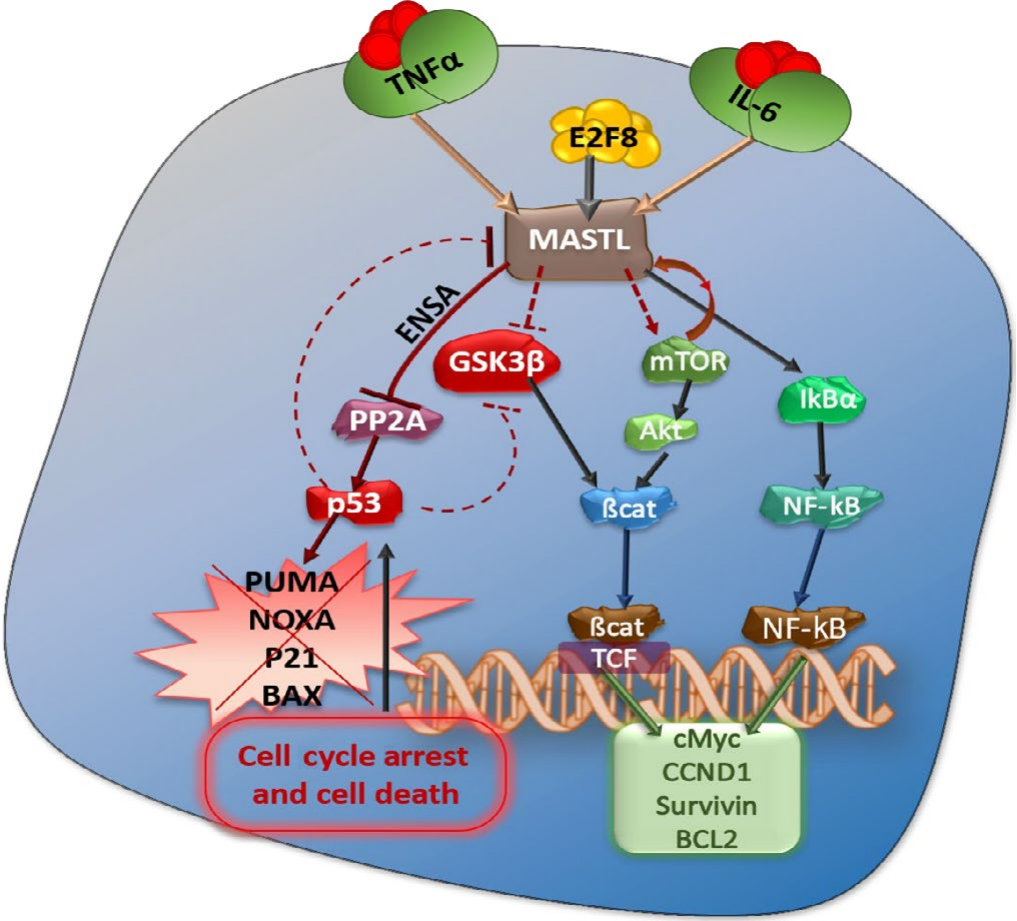
A summary of signaling networks of MASTL kinase in cancer

Furthermore, Nagel R et al showed that MASTL can be a therapeutic target for radiosensitization of non–small cell lung cancer (NSCLC).^24^, ^66^ Knockdown of MASTL expression induced radiosensitization in a panel of NSCLC cells, but not in the primary human fibroblasts. Recently, our group also demonstrated that MASTL is upregulated in CRC and its expression associates with the clinicopathological parameters and overall survival in CRC patients. MASTL mediates its effects through regulation of Wnt/β‐catenin signaling in colon cancer progression and resistance to anticolorectal cancer (CRC) therapy^24^ (Figure 1). Similarly, Wang et al demonstrated that MASTL upregulation correlates with cancer progression and tumor recurrence after initial cancer therapy in the recurrent tumors of the head and neck squamous cell carcinoma patients. MASTL knockdown in recurrent tumor cells resensitized their response to cancer therapy and potentiated cancer cells to cell death in chemotherapy. An overview of the potential role of MASTL in different types of cancers is summarized in Table 1.

**Table 1:** Overexpression of MASTL kinases in wide variety of cancers, making them as an attractive targets

S.No.	Cancer	Mechanism	References
1	Breast	PI3K/Akt/mTOR and p38 kinase signaling	Vera et al eLife (2015); Alvarez‐Fernández (2018)
2	Head and neck squamous cell carcinoma (SCC)	Akt signaling	Wang et al. (2014)
3	Gastric	Akt signaling/ EMT	Sun et al (2017); Maroto et al (2018)
4	Colon	β‐catenin/Wnt signaling and Akt	Uppada et al (2018); Vera et al eLife (2015)
5	Prostate	Akt signaling	Wangetal. (2014)
6	Thyroid		Cettietal. (2019)
7	Hepatocarcinoma	IL‐6 and TNFa/NFkB signaling	Cao et al. (2019)

Importantly, even though kinase activity of MASTL may play a role in transformation, MASTL has effects outside of mitosis and beyond regulation of PP2A‐B55 including control of the normal DNA replication timing, regulation of AKT/mTOR and Wnt/β‐catenin oncogenic kinase signaling, centrosome amplification and CIN.^24^, ^54^ MASTL overexpression promotes cell transformation in breast and colon cancer cells through hyperphosphorylation of the oncogenic kinase AKT or Wnt/β‐catenin signaling.^24^, ^54^ They demonstrated an indirect mechanism, independent of endosulfines, whereby S473 phosphorylation of Akt is increased through GSK3β‐dependent degradation of the S473 PHLPP phosphatase.^54^ Our studies also supported the observation that MASTL induces Wnt/β‐catenin signaling in colon cancer by regulating glycogen synthase kinase‐3 beta (GSK3β) phosphorylation.^24^ However, whether GSK3β dephosphorylation is dependent on PP2A remains unclear. In that context, Chu et al demonstrated that PP2A is involved in GSK3β dephosphorylation at S9,^67^ thus suggesting that this signaling cascade could still be partly dependent on PP2A‐B55 inhibition.^67^, ^68^ PP2A‐B55 has also been found to be a negative regulator of the β‐catenin phosphorylation and interferes with Wnt signaling.^69^ Similar to our group, Rogers et al also showed that MASTL overexpression leads to significant disruption to the members of the Wnt‐signaling pathway, including increased phosphorylation of β‐catenin and mislocalization of E‐cadherin.^23^, ^25^ However, the mechanism for MASTL regulation of GSK3β/Wnt signaling is still poorly understood and should be investigated in future studies. Overall, the above studies represent an alluring possibility that MASTL may have other substrates beyond ENSA and regulation of PP2A‐B55. In support of this hypothesis, it has previously been shown that Rim15p, the orthologue of MASTL in yeast, phosphorylates additional substrates, including the nutrient‐responsive transcription factors Msn2p/4p and Hsf1p, during starvation.^70^ Rim15 negatively regulates TORC1 (mTOR in humans) signaling under nutrient stress conditions, thus suggesting the possibility that MASTL may directly regulate the mTOR pathway in human cells. In future studies, it will be important to further tease apart the specific signaling pathways and cross‐talk to determine the specific genetic background and biomarkers involved in MASTL‐mediated regulation of oncogenesis.

## 4 REGULATION OF MASTL KINASE DURING CANCER PROGRESSION

Despite the well documented role of the MASTL in cancer progression and malignancy, its regulation remains largely unknown. Recent studies have provided evidence for the transcriptional regulation of MASTL expression during oncogenesis. A recent publication further revealed the role of proinflammatory cytokines tumor necrosis factor alpha (TNF‐α) or interleukin‐6 (IL‐6) in regulating the mRNA and protein expression of MASTL.^63^ Notably, these cytokines promoted trimethylation of histone H3K4 to facilitate chromatin accessibility at the MASTL promoter to promote nuclear factor kappa‐light‐chain‐enhancer of activated B cells (NF‐κB)‐induced MASTL transcription in liver cancer.^63^ In addition, inhibiting NF‐κB activity influenced MASTL mRNA expression, suggesting a role for NF‐κB in this process; however, details of this regulation are still unknown. Yet another transcription factor, E2F Transcription Factor 8 (E2F8), increases MASTL transcription via binding to its promoter.^71^ Of interest, E2F8 overexpression alleviates cisplatin‐induced cell apoptosis in MCF‐7 cells by shortening G2‐M arrest and promoting mitotic entry, the effect of which was largely abrogated by inhibiting MASTL. Further studies are needed to understand the precise signaling pathways and identify the cross‐talk regulation to determine regulation of MASTL in oncogenesis.

## 5 PHARMACOLOGIC TARGETING OF MASTL ACTIVITY IN CANCER THERAPY

As mentioned earlier, targeting of mitosis has been considered an attractive therapeutic strategy for selective anticancer treatment.^72^, ^73^ Considering its key significance in cancer progression and therapy resistance, recent years have witnessed growing interest in targeting MASTL as a therapeutic target for cancer therapy. Current studies have also demonstrated a possibility for targeting MASTL in therapy with DNA damaging agents including cisplatin, radiotherapy, and 5‐FU in several cancer types.^24^, ^52^, ^53^, ^66^ Therapeutic opportunities will depend on specific genetic backgrounds and tumor types. Several recent studies suggest that MASTL may play important role in regulating normal DNA replication timing,^11^ stimulating oncogenic AKT kinase activity,^10^ and recovery from premitotic DNA damage checkpoint arrest.^12^ MASTL is commonly overexpressed in several cancer types including colon, oral, and breast cancer^10^ and associated with cancer progression.^13^ MASTL is known to regulate cell proliferation, tumor growth, and metastasis in vivo in breast, thyroid, and colon cancer cells.^24^, ^25^, ^54^, ^74^ Inhibition of MASTL in colon cancer cells induced chemosensitivity to 5‐FU with downregulation of Survivin and Bcl‐xL expression, whereas MASTL depletion in breast cancer cells enhanced the radiosensitivity with increased PP2A activity. Together, these data suggest that MASTL inhibition possesses strong potential for cancer therapy with small molecule inhibitors.

Chemotherapy has dominated cancer therapeutics for a long time, but recently kinase inhibitors have also been proven to be efficient for cancer treatment. More than 25 kinase inhibitors have been approved for cancer therapy, and numerous others are under clinical trials.^75^, ^76^ However, in comparison to the other kinases such as PLK1 and Aurora kinases, MASTL is less studied. To date, there is a single compound named Greatwall Kinase Inhibitor‐1 (GKI‐1), which has been identified as a first line inhibitor against MASTL.^77^, ^78^ GKI‐1 interferes with full‐length human Gwl activity in vitro and is effective in altering cell viability by inhibiting the phosphorylation of ENSA/ARPP19 in HeLa cells, resulting in a decrease in mitotic events, mitotic arrest/cell death, and cytokinesis failure.^77^ Recent studies have developed new assays to screen kinase‐specific libraries for the search of potential MASTL inhibitors.^79^ To that end, there are few studies in which various drug databases have explored from virtual screening, both natural and synthetic compound sources.^78^ In 2018, Ammarah et al identified several compounds that may be inhibit MASTL kinase activity and potentially can be very useful. MASTL interaction with these compounds was further explored using molecular dynamics simulations. However, these inhibitors have not been tested directly yet against the enzyme itself, and thus cannot be described as either effective or to have high‐binding affinity.^78^ Altogether, this study identified potential inhibitors of human Gwl kinase from both natural and synthetic origins and calls for studying these compounds as potential drugs for cancer therapy. However, there is still a dearth of knowledge in the area of MASTL inhibition and needs to be addressed in near future.

## 6 CONCLUSION

Cell cycle division is regulated at various checkpoints, which are surveillance mechanisms to assure the precise chronological and spatial coordination of the cell cycle events. Spindle assembly checkpoint failure promotes centrosome amplification that interferes with the fidelity of correct chromosome segregation. Consequently, studies on cell cycle checkpoints are a very important area for further research for understanding the mechanisms of chromosomal stability and genome maintenance, as they have direct impact on the oncology field. MASTL kinase is the crucial mitotic regulator required for the maintenance of chromosomal stability. In addition, it also plays an important oncogenic role in regulating AKT, Wnt/β‐catenin signaling, DNA cohesion, and indirect role in the regulation of DNA damage response.^24^, ^54^, ^61^, ^65^ Upregulation of MASTL kinase in different kinds of cancer has been functionally associated with oncogenic transformation through the hyperactivation of oncogenic kinase AKT‐ or Wnt/β‐catenin signaling and resistance to anticancer therapy. These findings reveal that MASTL kinase represents an attractive druggable target in cancer by modulating the activity of key oncogenic signaling pathways associated with chemoresistance, onset of distant metastases, and poor patient outcome. Further studies are required to unearth complicated roles of MASTL kinase in tumorigenesis, and to understand the resistance mechanisms or possible synergies with other therapeutic strategies. Exploring the detailed molecular mechanisms of MASTL kinase will bring great rewards in understanding cell cycle control as well as add a promising new area of anticancer drug development. The small molecule compounds that can specifically inhibit this kinase will be of immense importance in cancer therapy.

## CONFLICT OF INTEREST

The authors declare no potential conflicts of interest.
